# Associations between sugar-sweetened beverage consumption, moderate-to-vigorous-intensity physical activity levels, and depressive symptoms: a cross-sectional study based on Tibetan university students at high altitude in China

**DOI:** 10.3389/fnut.2025.1582167

**Published:** 2025-05-29

**Authors:** Zhen Zhang, Nana Tang, Mengjin Yao, Tao Li

**Affiliations:** ^1^School of Physical Education, Huanghuai University, Zhumadian, China; ^2^College of Education, Tibet University, Lhasa, China

**Keywords:** sugar-sweetened beverage, moderate-to-vigorous-intensity physical activity, depressive symptoms, university students, Tibetan, high altitude

## Abstract

**Background:**

Depressive symptoms are spreading globally with increasing life stress and have become an important public health issue. However, fewer studies have been conducted on the association between sugar-sweetened beverages (SSBs) consumption, moderate-to-vigorous-intensity physical activity (MVPA), and depressive symptoms among Tibetan university students at high altitudes. The present study may provide a reference for the mental health development of Tibetan university students at high altitudes.

**Methods:**

In this study, SSBs consumption, MVPA time objectively measured by Actigraph GT3X+ accelerometer, and depressive symptoms were assessed in 1,062 university students aged 19–22 years from two universities in Lhasa, Tibet, and Xining, Qinghai, China, using stratified randomized whole-cluster sampling. The methods of chi-square test, logistic regression analysis, and ordered logistic regression analysis with a generalized linear model were used to analyze the association between SSBs consumption, MVPA levels, and the presence of depressive symptoms.

**Results:**

It was found that the proportions of Tibetan university students at high altitudes in China with SSBs 3–5 times/week and ≥6 times/week were 24.3% and 20.3%, respectively. The proportion of MVPA >60 min/day was only 5.6%. The prevalence of depressive symptoms was 37.5%. Ordered logistic regression analysis adjusted for covariates showed that, in general, for those with SSBs ≤2 times/week and MVPA >60 min/day, there was a positive association between those with SSBs ≥6 times/week and MVPA 30–60 min/day and the occurrence of depressive symptoms (OR = 5.92, 95% CI: 1.94–18.10). Those with SSBs ≥6 times/week and MVPA <30 min/day were also positively associated (*P* < 0.001) with the occurrence of depressive symptoms (OR = 5.91, 95% CI: 2.19–15.94).

**Conclusions:**

The prevalence of depressive symptoms among Tibetan university students at high altitudes in China is concerning. Higher SSB consumption and lower MVPA were associated with a higher prevalence of depressive symptoms. The findings of this study may provide necessary references and lessons for the government and educational departments to develop public health and educational measures for university students in high-altitude areas.

## 1 Introduction

It has been reported that with changing lifestyles, prolonged static behaviors, and decreasing levels of physical activity, symptoms of depression among university students are spreading globally, and its prevalence is showing a continuing trend of increase and has become an important mental health problem worldwide (1). Depressive symptoms, as a pre-existing state of depression, should receive timely attention and concern to reduce the occurrence of depressive symptoms. On the contrary, if depressive symptoms do not receive timely attention and guidance, they will develop into more serious depression, which will have an important negative impact on the physical and mental health development and academic achievement of university students. A meta-analysis involving 100,187 university students showed that the prevalence of depressive symptoms among university students averaged 33.6%, with the highest prevalence of 40.1% in Africa, followed by 54.2% in low- and middle-income countries (2). Surveys show that 31.5%−36.0% of university students in the United States have varying degrees of depressive symptoms, and this figure is continuing to rise (3). The prevalence of depressive symptoms among university students in China is also high and shows a trend of low aging, which has an important negative impact on the academic performance and future achievements of university students. According to the survey, the prevalence of depressive symptoms among university students in China is 46.40%, of which the rates of mild, moderate, moderately severe, and severe are 28.5%, 10.1%, 7.3%, and 0.6%, respectively (4). Studies have shown that patients with depressive symptoms often have problems such as decreased sleep quality and social disorders, which, if not attended to promptly will lead to non-suicidal self-injurious behaviors, depression, and, in severe cases, suicidal behaviors (5). It is noteworthy that previous studies on university students' depressive symptoms have mainly focused on plain areas, while fewer studies have been conducted on university students' depressive symptoms in high-altitude areas. Previous studies have confirmed that the prevalence of depressive symptoms in high-altitude areas is relatively higher than that in plain areas due to the influence of factors such as altitude and level of economic development (6). Therefore, it is necessary to investigate and analyze depressive symptoms among Tibetan university students at high altitudes.

At present, it has become an indisputable fact that university students' SSB consumption is increasing year by year and MVPA levels are decreasing year by year, which has an important negative impact on the physical and mental health development of university students (7, 8). Surveys show that 14% of Americans' daily calories come from added sugar, exceeding the World Health Organization's 10% recommendation (9). It is also of concern that more than half of the intake of added sugars comes from SSB consumption (10). Excessive consumption of SSBs leads to an increased risk of chronic diseases such as obesity, dental caries, diabetes, and hypertension (11). China, a developing country, is no exception, and surveys have shown that SSB consumption among Chinese university students continues to increase (12). Studies have shown that increased consumption of SSBs in university students leads to obesity, which negatively impacts physical and mental health development (13). It has also been shown that increased consumption of SSBs leads to a decrease in brain function, which will also lead to various psychological problems (14). In addition, some studies confirm that lower MVPA levels are also a significant contributor to the development of psychological problems (15). The decrease in MVPA levels is more related to the increasing time spent on online video screen behaviors, which leads to less time spent offline socializing, and is an important reason for the occurrence of various types of psychological problems (16). However, previous studies have focused on university students in plains areas, and there are fewer research studies investigating SSB consumption and MVPA for university students in high-altitude areas, especially fewer studies related to physical activity measured using objective instruments.

The Tibetan Plateau region of China is one of the world's typical high-altitude areas. Tibetans have a long history of living in this region and have developed human morphology and physiological characteristics that are different from those typical of the plains (17). Studies have shown that Tibetans at high altitudes have formed a broad and deep chest to adapt to the high altitude and lack of oxygen due to living in a cold and oxygen-poor environment for a long time (18). There are also studies confirming that the incidence of mental health problems among Tibetans at high altitudes is higher than in the plains, which should be emphasized and paid attention to (19–21). Other studies have shown that in recent years, the consumption of SSBs among Tibetans at high altitudes has continued to increase, while at the same time, the level of physical activity has shown a decreasing trend, which hurts physical and mental health, and should be given sufficient attention and concern (22). For this reason, this study conducted a questionnaire survey on SSB consumption and depressive symptoms and an objective assessment of MVPA with Actigraph GT3X+ accelerometer on 1062 Tibetan university students at high altitudes in China. The aim was to analyze the association between SSBs consumption and MVPA with depressive symptoms among Tibetan university students at high altitudes in China, to better promote the physical and mental health development of Tibetan university students at high altitudes.

## 2 Methods

### 2.1 Study participants and procedure

Participants in this study were drawn from two universities in Lhasa, Tibet, and Xining, Qinghai in the high-altitude region of China. The participant sampling process consisted of the following procedures. First, a convenience sampling method was used to select one university with more Tibetan university students in Lhasa, Tibet, and Xining, Qinghai, a high-altitude region of China, respectively, as the survey school for this study. Second, three teaching classes were randomly selected in a cluster of three from the first through fourth years of college in each university, and Tibetan university students in the classes who met the inclusion criteria for this study served as participants in this study. The specific inclusion criteria for Tibetan university students in this study were: both parents were Tibetan, the participants lived at a high altitude from birth to university, and volunteered to be evaluated in this study. In this study, a total of 1,092 Tibetan university students from 24 teaching classes in 2 universities were assessed and questionnaires were administered accordingly. After the assessment, 30 invalid data were excluded, including 11 invalid data from the physical activity test, 6 questionnaires with main demographic information missing, 12 questionnaires with a response rate lower than 80%, and 1 questionnaire that was broken. After the assessment, 1,062 valid data were finally returned, including 518 males and 544 females. The effective recovery rate of this questionnaire in this study was 97.25%. The mean age of the participants was (20.25 ± 1.03) years.

**Figure 1:**
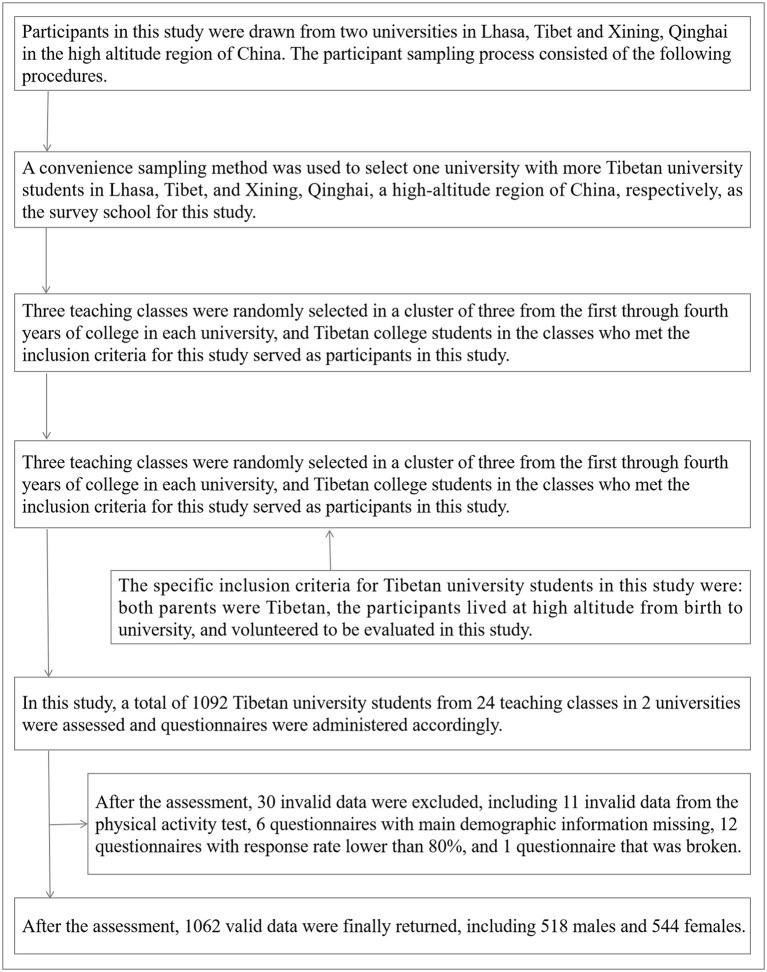
Participant extraction procedure for Tibetan university students in high-altitude areas of China.

All methods were performed by the Declaration of Helsinki. Written informed consent was obtained from the subjects before the investigation of this study, and the investigation was conducted voluntarily. This study was approved by the Ethics Committee of Tibet University (R8976356). Figure 1 shows the specific extraction process of participants in this study.

### 2.2 Sugar-sweetened beverages (SSBs)

SSB consumption of participants in this study was assessed using the beverage intake questionnaire (BEVQ-15) (23). The EVQ-15 assessment questionnaire consists of 15 items. The questionnaire mainly assessed participants' beverage intake behavior in the past 30 days. The questionnaire also significantly demonstrated sufficient construct validity and repeatability in this study to effectively assess SSB consumption among Tibetan university students at high altitudes. In previous studies, the BEVQ-15 was also used to investigate SSB consumption among Chinese university students (24). Participants in the survey were asked to fill in the type, frequency, and volume of beverages they consumed on average per day in the past 30 days. The types of beverages consumed included beer, wine, coffee, spirits, functional beverages, nut milk, carbonated beverages, fruit juices with added sugar, unsweetened beverages, tea with milk, milk with added sugar, and “other” beverages that were not mentioned in the survey assessment. For each type of beverage, participants filled in the frequency and amount of consumption according to their reality. Frequency was categorized as “never or <1 time/week”, “1 time/week”, “2–3 times/week”, “4–6 times/week “, “1 time/day”, “2 times/day”, “3 or more times/day”. The amount consumed each time was “ <6 ounces”, “8 ounces (1 cup)”, “12 ounces (1.5 cups)”, “16 ounces (2 cups),” “20 ounces (2.5 cups),” and “20 ounces and up.” The amount of each SSBs consumed by the participant and the total amount were calculated based on the formula established by BEVQ-15 (25). To facilitate this study's investigation of SSB consumption among Tibetan university students in high-altitude areas of China, the frequency and amount consumed were used to calculate the average daily consumption of university students, which was calculated as 8 ounces (1 cup) of approximately 250 mL each time. In this study, SSB consumption was categorized as “ ≤2 times/week”, “3–5 times/week”, and “≥6 times/week”.

### 2.3 Moderate-to-vigorous-intensity physical activity (MVPA) assessment

MVPA of participants in this study was assessed objectively using an Actigraph GT3X+ accelerometer (ActiGraph, FtWalton Beach, USA) (26). Actigraph GT3X+ by American Manufacturing Technologies, Inc. (27). The ability to monitor participants' steps, metabolic equivalents (METs), energy expenditure, activity intensity levels, and sleep duration, and analyze the data accordingly via Acti Graph ActiLife 6.1.4 software. The assessment method is currently recognized as an objective assessment of physical activity and the test method is relatively simple. The participant's name, date of birth, and gender were entered into the accelerometer via software before the test, and it was charged and distributed to the participant. Participants were instructed to wear the accelerometer on their right hip (28). Participants were required to wear the accelerometer continuously for 7 days, including 5 weekdays and 2 weekends. The accelerometers were required to be worn continuously except when the accelerometers were not worn in water, e.g., swimming, or bathing (29). The accelerometers were uniformly retrieved after 7 days and the data was processed through Acti Graph ActiLife 6.1.4 software. A minimum of 8 h of wear time in a day and a minimum of 40 min of non-zero data per hour were required. Valid data requires at least 2 valid weekdays and 1 valid weekend data. The average daily, school day, and weekend low physical activity (LPA) and MVPA levels were calculated based on the acceleration count thresholds (<2,800 counts/min for LPA, >2,800 counts/min for MVPA) established for Chinese university students (30). After obtaining the results of the accelerometer data, according to the World Health Organization (WHO) recommendation that adolescents should undergo MVPA for not <60 min per day, and the criteria for the division of MVPA time in related studies, the results of the accelerometer data were obtained (31). In this study, MVPA of Tibetan university students at high altitude was categorized into three groups: <30 min/day, 30–60 min/day, and >60 min/day.

### 2.4 Depressive symptom

Depressive symptoms in this study were assessed by the Center for Epidemiologic Studies Depression Scale (CES-D) (32). The scale includes indicators of depressive mood, interpersonal relationships, somatic symptoms, positive emotions, etc. The CES-D consists of 20 items and uses a four-point scale from 0 to 3 to score the frequency of related symptoms that have occurred in the last week among Tibetan university students at high altitudes. 0 = no (<1 day), 1 = occasionally (1–2 days), 2 = sometimes (3–4 days), and 3 = much (5–7 days); 4 of the questions required reverse scoring for a total score of 60, with higher scores indicating more severe depressive symptoms. A score of 0–16 was categorized as “No depressive symptoms”, 17–22 as “Mild depressive symptoms”, 23–27 as “Moderate depressive symptoms”, and 28–60 as “Severe depressive symptoms”. The questionnaires all have good reliability and validity (33, 34).

### 2.5 Covariates

The covariates in this study include urban or rural, only child, breakfast frequency, SES, BMI, screen time, sleep quality, and highly processed food. The covariates were all investigated using participant self-assessment questionnaires. Breakfast frequency was primarily assessed by how well participants ate breakfast each day for the past 7 days. It was categorized as ≤2 times/week, 3–5 times/week, and ≥6 times/week. Participants' SES was assessed by indicators such as parental literacy, parental occupation, and family income. The SES scores were calculated by a fixed formula after assigning values to the corresponding options (35). In this study, SES was categorized into Low (<25th), Medium (25th−75th), and High (>75th) based on percentile. BMI was assessed based on height and weight using the formula weight (kg)/height (m)^2^. Based on the classification criteria, BMI was categorized as Slimmer (<18.5 kg/m^2^), Normal (18.5–23.9 kg/m^2^), Overweight (24.0–27.9 kg/m^2^), Obese (≥28.0 kg/m^2^) (36). Screen time was assessed as the average time spent watching TV, tablet PCs, and cell phones in the past 7 days, and the frequency and duration of screen time were investigated, according to which the average screen time per day in the past 7 days was calculated. Screen time was categorized as ≤2 h/day and >2 h/day according to the relevant classification criteria, and sleep quality was assessed using the Pittsburgh Sleep Quality Index (PSQI). The PSQI was compiled by Dr. Buysse, a psychiatrist at the University of Pittsburgh, in 1989 (15). The scale is suitable for the assessment of sleep quality in the general population. The scale consists of 7 dimensions, each of which is rated on a scale of 0–3, with a total score of 0 to 21. Subjects scoring ≤5 were assessed as Good and >5 as Poor. In this study, highly processed food was defined as multi-ingredient processed recipe mixtures that are no longer processed to the extent of their original plant or animal source, such as margarine, sausage, processed cheese, and frozen pizzas (37). Participants were primarily assessed on the frequency of consumption in the past 7 days. This study was categorized as ≤2 times/week, 3–5 times/week, and ≥6 times/week.

### 2.6 Statistical analysis

The basic demographic characteristics and covariates of Tibetan university students in high-altitude areas of China were expressed as percentages, and comparisons between genders were made using chi-square tests. The basic demographic characteristics of depressive symptoms among Tibetan university students in high-altitude areas of China were also expressed as percentages, and comparisons between different levels of depressive symptoms were performed using chi-square tests. One-way analyses of SSBs consumption, MVPA, and depressive symptoms among Tibetan university students in high-altitude areas of China were compared using chi-square tests. The associations of SSBs consumption, MVPA, and depressive symptoms among Tibetan university students were analyzed by logistic regression. Model I was not adjusted for any covariates, Model II was adjusted for age, urban/rural, and whether or not being an only child based on Model I, and Model III was adjusted for breakfast frequency, SES, BMI, screen time, sleep quality, and ultra-processed food based on Model II. To further analyze in depth the association between SSBs consumption, MVPA, and depressive symptoms among Tibetan university students, ordered logistic regression analysis with the generalized linear model was conducted based on logistic regression analysis. The model-adjusted covariates in the analysis were age, urban/rural, being an only child, breakfast frequency, SES, BMI, screen time, sleep quality, and ultra-processed foods. Results are reported with ORs and 95% CIs, respectively.

## 3 Results

Table 1 shows that this study was a cross-sectional survey of SSBs consumption, MVPA, and depressive symptoms among 1,062 Tibetan university students at high altitudes in China. The results showed that the differences in Only child, Breakfast frequency, BMI, Sleep quality, SSB consumption, MVPA, and Depressive Symptoms among Tibetan university students at high altitude were statistically significant when compared (χ^2^-value of 43.658, 15.708, 81.379, 6.093, 13.258, 59.528, 14.094, *P* < 0.05).

**Table 1:** Basic demographic characteristics of Tibetan university students in high-altitude areas of China.

**Categories**	**Male**	**Female**	**χ^2^-value**	***P*-value**	**Total**
*N*	518	544			1,062
**Urban or Rural**			0.008	0.929	
Urban	125(24.1)	130(23.9)			255(24.0)
Rural	393(75.9)	414(76.1)			807(76.0)
**Only child**			43.658	<0.001	
Yes	172(33.2)	86(15.8)			258(24.3)
No	346(66.8)	458(84.2)			804(75.7)
**Breakfast frequency**			15.708	<0.001	
≤2 times/week	84(16.2)	52(9.6)			136(12.8)
3–5 times/week	151(29.2)	137(25.2)			288(27.1)
≥6 times/week	283(54.6)	355(65.3)			638(60.1)
**SES**			2.789	0.248	
Low	93(18.0)	82(15.1)			175(16.5)
Medium	352(68.0)	395(72.6)			747(70.3)
High	73(14.1)	67(12.3)			140(13.2)
**BMI**			81.379	<0.001	
Slimmer	48(9.3)	146(26.8)			194(18.3)
Normal	285(55.0)	304(55.9)			589(55.5)
Overweight	93(18.0)	38(7.0)			131(12.3)
Obese	92(17.8)	56(10.3)			148(13.9)
**Screen time**			2.019	0.155	
≤2 h/day	93(18.0)	82(15.1)			175(16.5)
>2 h/day	73(14.1)	67(12.3)			140(13.2)
**Sleep Quality**			6.093	0.014	
Good (≤5 points)	171(33.0)	142(26.1)			313(29.5)
Poor (>5 points)	347(67.0)	402(73.9)			749(70.5)
**Highly processed food**			2.969	0.227	
≤2 times/week	186(35.9)	222(40.8)			408(38.4)
3–5 times/week	210(40.5)	210(38.6)			420(39.5)
≥6 times/week	122(23.6)	112(20.6)			234(22.0)
**SSBs**			13.258	0.001	
≤2 times/week	287(55.4)	301(55.3)			588(55.4)
3–5 times/week	106(20.5)	152(27.9)			258(24.3)
≥6 times/week	125(24.1)	91(16.7)			216(20.3)
**MVPA**			59.528	<0.001	
<30 min/day	345(66.6)	471(86.6)			816(76.8)
30–60 min/day	131(25.3)	56(10.3)			187(17.6)
>60 min/day	42(8.1)	17(3.1)			59(5.6)
**Depressive symptoms**			14.094	0.003	
No depression symptoms	349(67.4)	315(57.9)			664(62.5)
Mild depression symptoms	87(16.8)	101(18.6)			188(17.7)
Moderate depression symptoms	79(15.3)	117(21.5)			196(18.5)
Severe depression symptoms	3(0.6)	11(2.0)			14(1.3)

N, Numbers; SES, Socio-economic status; BMI, Body mass index; SSBs, Sugar-sweetened beverages; MVPA, Moderate-to-vigorous-intensity physical activity.

The proportions of Tibetan university students in high-altitude areas of China with SSBs consumption of ≤2 times/week, 3–5 times/week, and ≥6 times/week were 55.4%, 24.3%, and 20.3%, respectively. The proportions of MVPA of <30 min/day, 30–60 min/day, and >60 min/day were 76.8%, 17.6%, and 5.6%, respectively. The percentages of MVPA for <30 min/day, 30–60 min/day, and >60 min/day were 76.8%, 17.6%, and 5.6%, respectively. The proportions of Depressive symptoms were 62.5%, 17.7%, 18.5%, and 1.3% for No Depression, Mild depression, Moderate depression, and Severe depression, respectively. Overall, the prevalence of Depressive symptoms among Tibetan university students in high-altitude areas of China was 37.5%.

Table 2 shows the comparison of basic demographic characteristics of depressive symptoms among Tibetan university students in high-altitude areas of China. The results showed that the prevalence of different levels of depressive symptoms among Tibetan university students in high-altitude areas of China was statistically significant when compared in terms of Gender, Only child, BMI, Screen time, Sleep quality, SSBs consumption, and MVPA (χ^2^-value of 14.094, 18.320, 19.760, 21.748, 168.331, 30.568, and 15.952, respectively, *P* < 0.05).

**Table 2:** Comparison of basic demographic characteristics of depressive symptoms among Tibetan university students in high-altitude areas of China.

**Categories**	**Depressive symptom**				**χ^2^-value**	***P*-value**
	**No**	**Mild**	**Moderate**	**Severe**		
***N***	664(62.52%)	188(17.70%)	196(18.46%)	14(1.32%)		
**Gender**					14.094	0.003
Male	349(52.6)	87(46.3)	79(40.3)	3(21.4)		
Female	315(47.4)	101(53.7)	117(59.7)	11(78.6)		
**Urban or Rural**					3.751	0.290
Urban	158(23.8)	54(28.7)	40(20.4)	3(21.4)		
Rural	506(76.2)	134(71.3)	156(79.6)	11(78.6)		
**Only child**					18.320	<0.001
Yes	189(28.5)	35(18.6)	30(15.3)	4(28.6)		
No	475(71.5)	153(81.4)	166(84.7)	10(71.4)		
**Breakfast frequency**					7.779	0.255
≤2 times/week	77(11.6)	23(12.2)	33(16.8)	3(21.4)		
3–5 times/week	172(25.9)	57(30.3)	54(27.6)	5(35.7)		
≥6 times/week	415(62.5)	108(57.4)	109(55.6)	6(42.9)		
**SES**					11.027	0.088
Low	94(14.2)	39(20.7)	40(20.4)	2(14.3)		
Medium	477(71.8)	129(68.6)	133(67.9)	8(57.1)		
High	93(14.0)	20(10.6)	23(11.7)	4(28.6)		
**BMI**					19.760	0.019
Slimmer	105(15.8)	44(23.4)	42(21.4)	3(21.4)		
Normal	389(58.6)	90(47.9)	107(54.6)	3(21.4)		
Overweight	83(12.5)	26(13.8)	18(9.2)	4(28.6)		
Obese	87(13.1)	28(14.9)	29(14.8)	4(28.6)		
**Screen time**					21.748	<0.001
≤2 h/d	217(32.7)	33(17.6)	44(22.4)	2(14.3)		
>2 h/d	447(67.3)	155(82.4)	152(77.6)	12(85.7)		
**Sleep quality**					168.331	<0.001
Good (≤5 points)	289(43.5)	12(6.4)	11(5.6)	1(7.1)		
Poor (>5 points)	375(56.5)	176(93.6)	185(94.4)	13(92.9)		
**Highly processed food**					5.400	0.494
≤2 times/week	249(37.5)	84(44.7)	69(35.2)	6(42.9)		
3–5 times/week	261(39.3)	71(37.8)	83(42.3)	5(35.7)		
≥6 times/week	154(23.2)	33(17.6)	44(22.4)	3(21.4)		
**SSBs**					30.568	<0.001
≤2 times/week	400(60.2)	95(50.5)	89(45.4)	4(28.6)		
3–5 times/week	152(22.9)	52(27.7)	52(26.5)	2(14.3)		
≥6 times/week	112(16.9)	41(21.8)	55(28.1)	8(57.1)		
**MVPA**					15.952	0.014
<30 min/day	493(74.2)	150(79.8)	160(81.6)	13(92.9)		
30–60 min/day	122(18.4)	33(17.6)	32(16.3)	0(0.0)		
>60 min/day	49(7.4)	5(2.7)	4(2.0)	1(7.1)		

N, Numbers; SES, Socio-economic status; BMI Body mass index; SSBs, Sugar-sweetened beverages; MVPA, Moderate-to-vigorous-intensity physical activity.

Table 3 shows the univariate analysis of SSBs consumption, MVPA, and depressive symptoms among Tibetan university students at high altitudes in China. The results showed that the prevalence of moderated depression symptoms among male students was statistically significant (χ^2^-value of 6.618, *P* < 0.05) when compared with different SSB consumption. The difference in the prevalence of severe depression symptoms was also statistically significant (χ^2^-value of 10.918, *P* < 0.01) when compared to the different SSB consumption aspects in female students. Overall, the prevalence of their moderate depression symptoms and severe depression symptoms compared to each other in terms of different SSB consumption, the differences were statistically significant (χ^2^-value of 7.966, 11.367, *P* < 0.05). In terms of MVPA, the prevalence of depressive symptoms compared to each other, none of the differences were statistically significant.

**Table 3:** Univariate analysis of SSBs consumption, MVPA, and depressive symptoms among Tibetan university students at high altitudes in China.

**Categories**	***N***	**Depressive Symptoms**											
		**No**			**Mild**			**Moderate**			**Severe**		
		***N*** **(%)**	χ^2^**-value**	***P*** **-value**	***N*** **(%)**	χ^2^**-value**	***P*** **-value**	***N*** **(%)**	χ^2^**-value**	***P*** **-value**	***N*** **(%)**	χ^2^**-value**	***P*** **-value**
**Male**													
**SSBs**													
≤2 times/week	287	208(72.5)	1.817	0.403	46(16.0)	0.196	0.907	33(11.5)	6.618	0.037	0(0.0)	4.117	0.128
3–5 times/week	106	69(65.1)			19(17.9)			17(16.0)			1(0.9)		
≥6 times/week	125	72(57.6)			22(17.6)			29(23.2)			2(1.6)		
**MVPA**													
<30 min/day	345	229(66.4)	0.479	0.787	61(17.7)	1.344	0.511	53(15.4)	1.002	0.606	2(0.6)	3.058	0.216
30–60 min/day	131	87(66.4)			22(16.8)			22(16.8)			0(0.0)		
>60 min/day	42	33(78.6)			4(9.5)			4(9.5)			1(2.4)		
**Female**													
**SSBs**													
≤2 times/week	301	192(63.8)	3.407	0.182	49(16.3)	1.611	0.447	56(18.6)	2.769	0.251	4(1.3)	10.918	0.004
3–5 times/week	152	83(54.6)			33(21.7)			35(23.0)			1(0.7)		
≥6 times/week	91	40(44.0)			19(20.9)			26(28.6)			6(6.6)		
**MVPA**													
<30 min/day	471	264(56.1)	2.288	0.319	89(18.9)	1.444	0.486	107(22.7)	4.212	0.122	11(2.3)	1.700	0.428
30–60 min/day	56	35(62.5)			11(19.6)			10(17.9)			0(0.0)		
>60 min/day	17	16(94.1)			1(5.9)			0(0.0)			0(0.0)		
**Total**													
**SSBs**													
≤2 times/week	588	400(68.0)	4.625	0.099	95(16.2)	1.581	0.454	89(15.1)	7.966	0.019	4(0.7)	11.367	0.003
3–5 times/week	258	152(58.9)			52(20.2)			52(20.2)			2(0.8)		
≥6 times/week	216	112(51.9)			41(19.0)			55(25.5)			8(3.7)		
**MVPA**													
<30 min/day	816	493(60.4)	2.667	0.264	150(18.4)	2.800	0.247	160(19.6)	4.728	0.094	13(1.6)	2.988	0.225
30–60 min/day	187	122(65.2)			33(17.6)			32(17.1)			0(0.0)		
>60 min/day	59	49(83.1)			5(8.5)			4(6.8)			1(1.7)		

N, Numbers; SSBs, Sugar-sweetened beverages; MVPA, Moderate-to-vigorous-intensity physical activity.

Table 4 shows the logistic regression analysis of SSBs consumption, MVPA, and depressive symptoms among Tibetan university students at high altitudes in China. The results showed that after adjusting for the relevant covariates, overall, with SSBs consumption ≤2 times/week as the reference group, there was a positive association between those Tibetan university students with SSBs consumption ≥6 times/week (OR = 1.60, 95% CI: 1.09–2.34) and the occurrence of depressive symptoms (*P* < 0.05). Using MVPA >60 min/day as the reference group, there was also a positive association between MVPA of 30–60 min/day (OR = 3.51, 95% CI: 1.54–8.02) and MVPA of <30 min/day (OR = 4.25, 95% CI: 1.97–9.20) with the occurrence of depressive symptoms (*P* < 0.01).

**Table 4:** Logistic regression analysis of SSBs consumption, MVPA, and depressive symptoms among Tibetan university students at high altitudes in China.

**Categories**		**Model I**		**Model II**		**Model III**	
		**OR (95% CI)**	***P -*** **value**	**OR (95% CI)**	***P -*** **value**	**OR (95% CI)**	***P -*** **value**
**Male**							
SSBs	≤2 times/week	1.00		1.00		1.00	
	3–5 times/week	1.41(0.88–2.27)	0.156	1.42(0.88–2.29)	0.153	1.33(0.75–2.38)	0.331
	≥6 times/week	1.88(1.21–2.91)	0.005	1.95(1.25–3.04)	0.003	1.05(0.60–1.83)	0.866
MVPA	>60 min/day	1.00		1.00		1.00	
	30–60 min/day	2.15(0.92–5.04)	0.078	2.23(0.95–5.26)	0.066	3.51(1.33–9.29)	0.011
	<30 min/day	2.15(0.97–4.80)	0.061	2.22(0.99–4.98)	0.054	3.82(1.53–9.55)	0.004
**Female**							
SSBs	≤2 times/week	1.00		1.00		1.00	
	3–5 times/week	1.46(0.99–2.18)	0.059	1.46(0.98–2.18)	0.062	1.47(0.95–2.28)	0.081
	≥6 times/week	2.25(1.40–3.62)	0.001	2.28(1.41–3.69)	0.001	2.35(1.35–4.07)	0.003
MVPA	>60 min/day	1.00		1.00		1.00	
	30–60 min/day	9.60(1.19–77.73)	0.034	10.83(1.32–88.64)	0.026	7.40(0.87–63.07)	0.067
	<30 min/day	12.55(1.65–95.38)	0.015	12.98(1.69–99.54)	0.014	9.70(1.23–76.71)	0.031
**Total**							
SSBs	≤2 times/week	1.00		1.00		1.00	
	3–5 times/week	1.48(1.10–2.01)	0.011	1.47(1.08–2.00)	0.013	1.37(0.97–1.92)	0.072
	≥6 times/week	1.94(1.41–2.67)	<0.001	2.02(1.46–2.78)	<0.001	1.60(1.09–2.34)	0.017
MVPA	>60 min/day	1.00		1.00		1.00	
	30–60 min/day	2.96(1.37–6.40)	0.006	3.18(1.46–6.92)	0.004	3.51(1.54–8.02)	0.003
	<30 min/day	3.64(1.77–7.50)	<0.001	3.69(1.78–7.64)	<0.001	4.25(1.97–9.20)	<0.001

SSBs, Sugar-sweetened beverages; MVPA, Moderate-to-vigorous-intensity physical activity. Model I was not adjusted for any covariates, Model 2 was adjusted for age, urban/rural, and whether or not the child was an only child based on Model I, and Model 3 was adjusted for breakfast frequency, SES, BMI, screen time, sleep quality, and ultra-processed foods based on Model 2.

Table 5 shows the ordered logistic regression analyses of SSBs consumption, MVPA, and depressive symptoms among Tibetan university students at high altitudes in China. Overall, the results showed that with SSB consumption ≤2 times/week and MVPA >60 min/day as the reference group, there was a positive correlation between SSB consumption ≤2 times/week and MVPA <30 min/day and the occurrence of depressive symptoms (OR = 3.41,95% CI: 1.30–8.95) (*P* < 0.05), were positively associated with the occurrence of depressive symptoms (*P* < 0.05). SSB consumption 3–5 times/week and MVPA 30–60 min/day were positively associated with the development of depressive symptoms (OR = 5.61,95% CI: 1.81–17.37) (*P* < 0.01). SSB consumption 3–5 times/week and MVPA <30 min/day were positively associated (*P* < 0.01) with the development of depressive symptoms (OR = 4.34, 95% CI: 1.62–11.62). SSB consumption ≥6 times/week and MVPA 30–60 min/day were positively associated (*P* < 0.01) with the development of depressive symptoms (OR = 5.92, 95% CI: 1.94–18.10). SSB consumption ≥6 times/week and MVPA <30 min/day were positively associated (*P* < 0.001) with the occurrence of depressive symptoms (OR = 5.91, 95% CI: 2.19–15.94).

**Table 5:** Ordered logistic regression analysis of SSBs consumption, MVPA, and depressive symptoms among tibetan university students in high altitude areas of China.

**Genders**	**Categories**		**Ordered logistic regression**	
	**SSBs**	**MVPA**	**OR (95% CI)**	***P-*** **value**
Male	≤2 times/week	>60 min/day	1.00	
		30–60 min/day	1.64(0.49–5.45)	0.422
		<30 min/day	2.79(0.93–8.40)	0.068
	3–5 times/week	>60 min/day	3.00(0.41–22.18)	0.282
		30–60 min/day	5.14(1.39–19.05)	0.014
		<30 min/day	2.71(0.84–8.70)	0.095
	≥6 times/week	>60 min/day	2.00(0.29–13.62)	0.479
		30–60 min/day	5.67(1.63–19.76)	0.006
		<30 min/day	4.04(1.28–12.72)	0.017
Female	≤2 times/week	>60 min/day	1.00	
		30–60 min/day	2.92(0.32–26.89)	0.345
		<30 min/day	4.33(0.53–35.73)	0.173
	3–5 times/week	>60 min/day	–	–
		30–60 min/day	7.00(0.67–72.86)	0.104
		<30 min/day	6.19(0.74–51.73)	0.092
	≥6 times/week	>60 min/day	–	–
		30–60 min/day	7.00(0.57–86.32)	0.129
		<30 min/day	9.40(1.11–79.93)	0.040
Total	≤2 times/week	>60 min/day	1.00	
		30–60 min/day	1.96(0.69–5.59)	0.207
		<30 min/day	3.41(1.30–8.95)	0.013
	3–5 times/week	>60 min/day	1.69(0.35–8.28)	0.517
		30–60 min/day	5.61(1.81–17.37)	0.003
		<30 min/day	4.34(1.62–11.62)	0.003
	≥6 times/week	>60 min/day	1.77(0.28–11.08)	0.541
		30–60 min/day	5.92(1.94–18.10)	0.002
		<30 min/day	5.91(2.19–15.94)	<0.001

SSBs, Sugar-sweetened beverages; MVPA, Moderate-to-vigorous-intensity physical activity. The ordered logistic regression analysis model was adjusted for the following covariates: age, urban/rural, being an only child, breakfast frequency, SES, BMI, screen time, sleep quality, and ultra-processed foods.

## 4 Discussion

This study found that the prevalence of psychological symptoms among Tibetan university students in high-altitude areas of China was high, and their overall situation was not optimistic. In addition, increased consumption of SSBs among Tibetan university students at high altitudes in China was associated with a higher prevalence of depressive symptoms, and there was a positive correlation between the two. There was a negative correlation between MVPA and the prevalence of depressive symptoms among Tibetan university students at high altitudes in China, i.e., the longer the duration of MVPA, the lower the prevalence of depressive symptoms among Tibetan university students. The findings of this study may assist with the targeted intervention of depressive symptoms among Tibetan university students in high-altitude areas in China, and enrich the literature on mental health interventions for university students in high-altitude areas. It also provides information for the government and education department to formulate public health policies for schools in high-altitude areas.

It is an indisputable fact that the consumption of SSBs by university students continues to increase. At the same time, physical activity is also declining, especially MVPA levels, which are important for the health of university students, are declining, and this harms the mental health of university students (38). The results of this study show that the proportion of Tibetan university students in China's high-altitude areas with SSBs consumption ≥6 times/week is 20.3%, a result that is relatively high compared to other studies. It can be seen that the availability of SSB consumption for Tibetan university students living in high-altitude areas is gradually increasing with the rapid development of the logistics industry as a result of the economic development of the Tibetan region in China. This leads to the fact that university students can buy all kinds of SSBs consumption at any time, and this reason is an important factor contributing to the higher SSB consumption by Tibetan university students in high-altitude areas. The results of this study also showed that 76.8% of Tibetan university students at high altitudes in China had MVPA <30 min/day, while only 5.6% had MVPA >60 min/day. This shows that more than 90% of Tibetan university students at high altitudes do not meet the standards recommended by the World Health Organization. The reason for this is firstly related to the harsh natural environment at high altitude, and the lack of oxygen at high altitude restricts strenuous exercise, which is an important reason for the low MVPA. In addition, longer sunshine hours, high outdoor temperatures, and strong UV rays are also one of some important reasons for lower MVPA. This study also showed that overall, the prevalence of depressive symptoms among Tibetan university students in high-altitude areas of China was 37.5%. This result was higher than the finding of 36.5% for university students in the plains region of China (13). It is also higher than the global prevalence of depressive symptoms among adolescents, which is 34% (39). It can be seen that the mental health status of Tibetan university students in high-altitude areas in China is not optimistic. Relevant investigations and studies should be conducted to address the causes of reducing the depressive symptoms of Tibetan university students in high-altitude areas.

The results of the present study also showed that there was a positive association between increased SSB consumption and the occurrence of depressive symptoms among Tibetan university students at high altitudes in China. Currently, there is a lack of clarity regarding the underlying mechanisms between SSB consumption and the development of depressive symptoms, and some possible explanations include the following. First, excessive SSB consumption is an important risk factor for the development of obesity, which is an important factor for the development of depressive symptoms, and this may be an important reason for the close association between increased SSB consumption and the development of depressive symptoms (40, 41). Secondly, most SSBs come in plastic packaging, which is prone to leaching phthalates. These are tiny particles that the body can't break down. They can even get into the bloodstream and reach the brain, potentially harming brain health and possibly contributing to depressive symptoms (42). Third, the excessive consumption of SSBs tends to coincide with the consumption of high-energy foods, and their poor dietary behavioral habits and reduced intake of fresh vegetables and fruits may also be an important risk factor for the development of depressive symptoms (43). Fourth, SSBs contain certain ingredients such as caffeine, which, when consumed in large quantities, can hurt sleep, and brain health, and is a significant contributor to the development of depressive symptoms (44). Subsequently, overconsumption of SSBs leads to increased levels of inflammation in the body, as well as changes in gut flora, all of which are strongly associated with depressive symptoms (45).

The present study also showed that there was also an association between reduced MVPA and elevated depressive symptoms among Tibetan university students in high-altitude areas of China. Past studies have also confirmed the association between MVPA and depressive symptoms for a variety of reasons (46). First, the reduction in MVPA was associated with prolonged video screen time, which is associated with the development of depressive symptoms, and prolonged video screen time is a risk factor for the development of depressive symptoms (47). Second, reduced MVPA is linked to a higher risk of developing obesity. And obesity, in turn, is connected to a range of psychological issues. Research has shown that university students who become obese are more likely to experience symptoms of depression and anxiety (16). Third, lower MVPA tends to cut into face-to-face socializing time, and keeping up a decent amount of in-person social interaction is tied to better mental health (48). This is because effective offline communication and interaction between peers or friends have a positive effect on relieving internalized stress. Additionally, decreased MVPA has been associated with sleep disorders, changes in gut flora, and increased inflammatory response, which are also important risk factors for the development of depressive symptoms (49, 50).

The present study has certain strengths and limitations. Strengths: First, to the best of our knowledge, this study analyzed for the first time the association between SSBs consumption and MVPA with depressive symptoms in Tibetan university students at high altitudes in China, providing information on the development of physical and mental health of Tibetan university students at high altitude. Second, MVPA in this study was measured objectively using an Actigraph GT3X+ accelerometer, which has a relatively high sample size, and the accuracy of data testing is another advantage of this study. However, this study also has some limitations. First, the SSB consumption investigated in this study is at the beginning of the school year in September, and due to the influence of the season, there is a certain deviation between its SSB consumption and the season, which inevitably affects the results. Secondly, the present study is a cross-sectional survey, which can only understand the correlation that exists between SSBs consumption and MVPA and depressive symptoms, but not the causal association, which is also a shortcoming of this study. Prospective cohort studies should be conducted in the future to better analyze the causal associations that exist between them. In addition, the covariates included in this study were limited, such as smoking and alcohol consumption that affected the symptoms of depression. A wider assessment should be conducted in future research to improve the rigor of the analysis results.

## 5 Conclusions

In conclusion, the depression symptoms of Tibetan university students in high-altitude areas in China are not optimistic, and there is an association between SSBs and MVPA and depression symptoms, with higher consumption of SSBs and lower MVPA being associated with a higher risk of depression symptoms. Because of this, it is important to emphasize the physical and mental health development of Tibetan university students in high-altitude areas of China, and to take necessary measures to limit the consumption of SSBs and increase the MVPA levels of Tibetan university students, to reduce the occurrence of depressive symptoms. The results of this study may provide necessary references and lessons for the government and educational departments to develop public health and educational measures for university students in high-altitude areas.

## Data Availability

The raw data supporting the conclusions of this article will be made available by the authors, without undue reservation.
